# Clinical and imaging features in the differentiation of pediatric pulmonary tuberculosis and pneumonia

**DOI:** 10.3389/fped.2026.1812951

**Published:** 2026-05-19

**Authors:** Xiaoxia Wang, Shaomei Huang, Hua Chen

**Affiliations:** 1 Department of Pediatrics, Guangzhou Chest Hospital, Guangzhou, China; 2 Department of Nontuberculous Mycobacteria, Guangzhou Chest Hospital, Guangzhou, China

**Keywords:** differential diagnosis, mycobacterium tuberculosis, pediatric, pneumonia, pulmonary tuberculosis

## Abstract

**Objective:**

To compare the clinical and Imaging characteristics between children with pediatric pulmonary tuberculosis (PTB) and children with pneumonia who were initially suspected of PTB, and to explore valuable indicators for differential diagnosis.

**Methods:**

This retrospective study enrolled pediatric inpatients initially suspected of PTB at Guangzhou Chest Hospital from March 2020 to May 2021. All participants were divided into the PTB group and the control group. Clinical characteristics, laboratory results, and chest computed tomography (CT) imaging findings were compared between the two groups. On the basis of univariate analysis, variables were further screened via the least absolute shrinkage and selection operator (LASSO) regression with reference to clinical significance. A stable and reliable predictive model was finally established using Firth penalized logistic regression.

**Results:**

A total of 63 children were enrolled in this study. Among them, 48 were finally diagnosed with PTB, including 23 cases with etiological confirmation and 25 cases with clinical diagnosis, which constituted the PTB group. The remaining 15 children were diagnosed with pneumonia and assigned to the control group. Compared with the control group, the PTB group had a significantly higher rate of tuberculosis contact history, longer pre-hospital symptom duration, and higher rates of pre-hospital antibiotic and systemic glucocorticoid use. In terms of chest CT manifestations, multiple pulmonary nodules, hilar/mediastinal/axillary lymphadenopathy, calcification, and fine linear or reticular opacities were significantly more common in the PTB group. The final predictive model was constructed based on the two most differentiating imaging features, namely multiple pulmonary nodules and hilar/mediastinal/axillary lymphadenopathy, and presented excellent diagnostic efficacy. The area under the receiver operating characteristic curve was 0.99. The corresponding sensitivity, specificity and overall accuracy were 93.75%, 100% and 95.24%, respectively.

**Conclusion:**

This study preliminarily developed an imaging model based on multiple pulmonary nodules and hilar/mediastinal/axillary lymphadenopathy, which may assist in distinguishing pediatric PTB from pneumonia. However, given the single-center, specialized-hospital setting of this study and the exclusion of certain patient subgroups, the generalizability of these findings is limited. Therefore, the conclusions require validation through future multicenter, large-scale prospective studies.

## 1 Introduction

Pulmonary tuberculosis (PTB), a chronic infectious disease caused by *Mycobacterium tuberculosis* (MTB) lung infection, remains a major global public health threat, and children are a highly vulnerable population. The World Health Organization (WHO) has reported a substantial burden of pediatric tuberculosis, which is frequently complicated by delayed diagnosis and unfavorable clinical outcomes (1). The diagnosis of pediatric PTB is challenging due to non-specific clinical manifestations that often overlap with common childhood respiratory diseases such as pneumonia (2). In addition, etiological confirmation is difficult in children because of the paucibacillary feature of this disease and difficulties in collecting qualified respiratory specimens. Clinicians therefore rely heavily on clinical, radiological and epidemiological evidence for diagnosis (3).

Such diagnostic uncertainty leads to a common clinical difficulty: distinguishing pediatric PTB from community-acquired pneumonia with tuberculosis-like manifestations. In clinical practice, these two diseases are easily confused with each other. Bidirectional misdiagnosis may cause disease progression, increase the risk of complications including miliary tuberculosis and intracranial tuberculosis, and adversely affect long-term prognosis. Therefore, identifying reliable clinical, laboratory, and imaging markers that can accurately distinguish between these entities at the point of initial suspicion is a critical unmet need in pediatric respiratory medicine.

In clinical practice at Guangzhou Chest Hospital, many children admitted with an initial suspicion of PTB are finally diagnosed to have pneumonia after systematic assessment, indicating a high rate of diagnostic overlap between the two diseases. Previous studies have separately summarized the clinical features of pediatric PTB and pneumonia. However, few studies have performed a targeted comparative analysis focusing on children with initial suspected of PTB, namely the population with prominent diagnostic confusion.

Therefore, this study aimed to compare clinical manifestations, laboratory indicators and chest imaging findings between children with diagnosed PTB and children initially suspected of PTB but finally diagnosed with pneumonia. The extracted differential indicators may assist clinicians in early identification of the two diseases, reduce the rate of misdiagnosis, and optimize clinical management strategies for affected children.

## 2 Subjects and methods

### 2.1 Study subjects

Children admitted to the Pediatric Department of Guangzhou Chest Hospital between March 2020 and May 2021 and initially suspected of PTB were enrolled in this study.

#### 2.1.1 Inclusion and exclusion criteria

Etiologically confirmed PTB was defined as a positive result for *Mycobacterium tuberculosis* nucleic acid testing, culture, or acid-fast bacilli smear (any single positive item). Clinically diagnosed PTB was determined based on clinical and imaging manifestations, significant improvements in symptoms and imaging findings after effective anti-tuberculosis therapy, and the exclusion of other competing diseases. All diagnoses referred to the national health industry standard *Diagnosis of Tuberculosis* (WS 288-2017) (4).

Inclusion criteria for the PTB group were as follows: (1) Meeting the above PTB diagnostic criteria; (2) No anti-tuberculosis treatment before admission.

The control group consisted of children initially suspected of PTB but finally diagnosed with pneumonia. Pneumonia diagnosis complied with the *Diagnostic Criteria for Community-Acquired Pneumonia in Children (2019 Edition)* (5). Additional inclusion requirements for the control group included: (1) No empirical anti-tuberculosis treatment after admission; (2) Significant absorption of pulmonary lesions on follow-up chest computed tomography (CT) after antibacterial treatment, and no evidence of PTB at the 6-month follow-up; (3) Absence of concurrent active tuberculosis, confirmed by negative results for sputum or gastric fluid acid-fast bacilli smear, negative *Mycobacterium tuberculosis* molecular testing and rapid culture, a tuberculin purified protein derivative (PPD) induration less than 10 mm, and a negative interferon-gamma release assay (IGRA).

Exclusion criteria for both groups were: (1) Presence of genetic or metabolic diseases; (2) Primary or secondary immunodeficiency, malignant tumors, or other severe chronic underlying disorders; (3) Rheumatic immune diseases or administration of biological immunosuppressive agents within one month before admission; (4) For the PTB group, patients with other concomitant pathogenic infections requiring targeted anti-infective treatment during hospitalization were excluded; for the control group, patients with fungal infection were excluded.

### 2.2 Study methods

#### 2.2.1 Data collection

Comprehensive clinical characteristic data of children in both groups were collected, including the following specific contents:

1. General information: Gender, age, height, weight, place of residence (urban/rural), history of tuberculosis exposure, duration of disease before admission, use of antibiotics and hormones before admission.
2. Clinical manifestations: Fever, cough, expectoration, shortness of breath, hemoptysis/blood—streaked sputum, chest pain, extrapulmonary clinical manifestations (such as digestive tract symptoms, headache and dizziness, disturbance of consciousness), and results of lung auscultation
3. Results of routine laboratory tests: White blood cell count, hemoglobin level, high-sensitivity C-reactive protein (hs-CRP), procalcitonin (PCT), total protein, albumin, alanine aminotransferase (ALT), aspartate aminotransferase (AST), PPD test results, blood IGRA results, and etiological test results, including pathogenic bacterial culture of sputum or bronchoalveolar lavage fluid, sputum or gastric juice acid-fast bacilli smear, rapid M. tuberculosis culture, M. tuberculosis molecular biological assay, Fungal culture, and detection of eight common respiratory pathogens.
4. Imaging examination results: chest CT findings. Bronchoscopy findings were documented for children with PTB who were complicated by tracheobronchial tuberculosis(TBTB). Head magnetic resonance imaging(MRI) findings were recorded for children with PTB who were complicated by Central Nervous System Tuberculosis(CNS-TB).

#### 2.2.2 Etiological detection

The following etiological examinations were performed in all children:Sputum (or bronchoalveolar lavage fluid) samples were collected for pathogenic bacterial and fungal culture;Sputum (or gastric juice) samples were collected for acid-fast bacilli smear, rapid M. tuberculosis culture (liquid method), and M. tuberculosis molecular biological detection;Venous blood was collected for detection of eight respiratory pathogens (including *Legionella pneumophila, Mycoplasma pneumoniae, Rickettsia burnetii, Chlamydia pneumoniae*, *influenza A virus*/ *influenza B virus* IgM, *parainfluenza virus*, *respiratory syncytial virus*, and *adenovirus*) and fungal serum antigen detection; During hospitalization, bronchoscopy was performed according to the children's condition, and bronchoalveolar lavage fluid was obtained for etiological detection (high-throughput sequencing was performed if necessary).

#### 2.2.3 Imaging assessment protocol

All chest CT scans were performed using the same 64-slice spiral CT scanner (Siemens Somatom Definition AS) with a unified protocol: tube voltage 100–120 kV, tube current 50–100 mAs, slice thickness 1.0 mm, reconstruction interval 0.5 mm, lung window (window width 1,500–2,000 HU, window level −600 to −700 HU) and mediastinal window (window width 300–400 HU, window level 30–50 HU) reconstruction. Two radiologists with more than 10 years of experience in chest imaging independently read the CT images in a blinded manner (unaware of the clinical diagnosis and laboratory results). The diagnostic criteria for key CT findings were as follows: (1) Lymphadenopathy: lymph node short-axis diameter ≥10 mm; (2) Multiple nodules: ≥3 pulmonary nodules with diameter <10 mm; (3) Fine linear/reticular opacities: thin linear or reticular shadows distributed in the pulmonary interstitium; (4) Patchy shadows with blurred edges: flocculent or sheet-like high-density shadows with unclear boundaries in the pulmonary parenchyma. Discrepancies in reading results were resolved through joint discussion by the two radiologists and a third senior radiologist. The inter-rater reliability was evaluated using the Kappa coefficient, and the Kappa values of all key CT findings were >0.8, indicating good consistency.

#### 2.2.4 Statistical analysis

All statistical analyses were conducted using R software (version 4.2.1). Continuous variables are presented as mean ± standard deviation or median with interquartile range according to their distribution. Categorical variables are expressed as frequencies and percentages. Between-group comparisons were performed using the independent-samples *t* test, Mann–Whitney U test, chi-square test, or Fisher exact test, as appropriate. A *P* value <0.05 was considered statistically significant. Descriptive data are reported to one decimal place, and *P* values less than 0.001 are presented as *P* < 0.001.

For multivariable predictive modeling, variables with *P* < 0.05 in univariate analysis were initially enrolled. To reduce overfitting and multicollinearity, variable selection was conducted via the least absolute shrinkage and selection operator (LASSO) regression. Two clinically meaningful and statistically stable predictors were finally screened and retained. Considering the limited and unbalanced sample size, multivariable regression analysis was performed using Firth penalized logistic regression. Bootstrap resampling with 1,000 iterations was used to obtain stable odds ratios (OR) and 95% confidence intervals (CI). Model diagnostic efficiency was assessed by the area under the receiver operating characteristic curve (AUC), sensitivity, specificity and overall accuracy.

## 3 Results

### 3.1 Comparison of general information between the two groups

A total of 63 children were enrolled in this study. Of these, 48 were ultimately diagnosed with PTB (23 etiologically confirmed and 25 clinically diagnosed) and constituted the PTB group; the remaining 15, diagnosed with pneumonia, formed the control group.

There were no statistically significant differences in gender, age, height, weight, and place of residence between the two groups (all *P* > 0.05), indicating good comparability. However, the number of children with a history of tuberculosis exposure, disease duration before admission >14 days, and use of antibiotics and hormones before admission in the PTB group was significantly higher than that in the pneumonia control group, with statistically significant differences (*P* < 0.05 or *P* < 0.001). The specific data are shown in Table 1.

**Table 1:** Baseline demographic and clinical history characteristics of pediatric patients with PTB vs. those with pneumonia initially suspected of PTB.

General Information	PTB group (*n* = 48)	Control group (*n* = 15)	*P*	OR (95%CI)
Gender (Male/Female) (cases)	24/24	8/7	0.82	0.88 (0.28, 2.73)
Age (years) [M (P25, P75)]	10.9 (8.0, 13.0)	8.5 (6.5, 12.5)	0.42	-
Height (dm) [M (P25, P75)]	14.5 (11.5, 15.8)	13.4 (11.2, 14.8)	0.81	-
Weight (kg) ($\bar{x}\pm s$)	31.4 ± 16.3	33.7 ± 15.9	0.65	-
Urban/Rural (cases)	35/13	10/5	0.64	1.35 (0.39, 4.69)
History of tuberculosis exposure [cases (%)]	26 (54.1)	3 (20.0)	0.02	4.76 (1.18,18.87)
Disease duration before admission >14 d [cases (%)]	38 (79.1)	7 (46.6)	0.02	4.34 (1.27,14.93)
Use of antibiotics and hormones before admission [cases (%)]	40 (83.3)	6 (40.0)	<0.001	7.50 (2.08,26.93)

-, Not statistically analyzed.

### 3.2 Comparison of clinical manifestations between the two groups

With the exception of fever accompanied by chills (*P* < 0.001), there were no significant differences between the groups in the incidence of other major respiratory symptoms (all *P* > 0.05). In terms of lung auscultation results, a statistically significant difference was observed between the two groups (*P* < 0.001). Regarding extrapulmonary manifestations, statistically significant differences were observed between the two groups (*P* < 0.05), except for findings related to digestive tract symptoms. The detailed clinical manifestations and their frequencies for both study groups are summarized in Table 2.

**Table 2:** Comparison of clinical manifestations, respiratory symptoms, lung auscultation findings, and extrapulmonary features between the PTB group and control group [cases (%)].

Clinical Manifestations	PTB Group (*n* = 48)	Control Group (*n* = 15)	*P*	OR (95%CI)
No respiratory symptoms	6 (12.5)	1 (6.6)	0.67	1.98 (0.23,16.87)
Respiratory symptoms	-	-	-	-
Fever	40 (83.3)	14 (93.3)	0.59	0.36 (0.04, 2.85)
Cough and expectoration	42 (87.5)	14 (93.3)	0.88	0.60 (0.08,4.55)
Shortness of breath	17 (35.4)	5 (33.3)	0.88	1.10 (0.34,3.52)
Hemoptysis/blood—streaked sputum	9 (18.7)	1 (6.6)	0.48	3.16 (0.38,26.10)
Chest pain	5 (10.4)	1 (6.6)	0.67	1.62 (0.19,13.85)
fever accompanied by chills	0 (0.0)	11 (73.3)	<0.001	0.02 (0.00, 0.15)
Fatigue, weight loss, night sweats	5 (10.4)	1 (6.6)	0.67	1.62 (0.19,13.85)
Lung auscultation	-	-	-	-
Abnormal	10 (20.8)	14 (93.3)	<0.001	0.02 (0.00,0.13)
Extrapulmonary manifestations	-	-	-	-
Digestive tract symptoms	8 (16.6)	3 (20.0)	0.33	0.80 (0.20,3.22)
Headache and dizziness	10 (20.8)	8 (53.3)	0.02	0.23 (0.07,0.77)
Disturbance of consciousness	5 (10.42)	0 (0.00)	<0.001	-

-, Not statistically analyzed.

### 3.3 Comparison of routine laboratory test results between the two groups

The PTB group had significantly lower proportions than the control group for: elevated ALT/AST (6.2% vs. 26.6%; *P* < 0.05) and PCT >0.5 ng/ml (0.0% vs. 86.6%; *P* < 0.001).

A comparison of the detailed laboratory findings between the two groups is presented in Table 3.

**Table 3:** Comparison of routine laboratory parameters, inflammatory markers, and etiological test results between children diagnosed with PTB and the control group.

Routine laboratory items	PTB group (*n* = 48)	Control group (*n* = 15)	*P*	OR (95%CI)
White blood cells (×10^9^ /L) [M (P25, P75)]	8.3 (6.1, 12.1)	11.3 (8.1, 13.3)	0.25	-
Hemoglobin (g/L) ($\bar{x}\pm s$)	112.1 ± 21.0	123.2 ± 15.8	0.07	-
hs-CRP (mg/L) [M (P25, P75)]	15.3 (3.7, 24.8)	22.2 (2.0, 48.4)	0.31	-
Elevated ALT or AST at admission [cases (%)]	3 (6.2)	4 (26.6)	0.03	0.18 (0.04, 0.82)
Albumin (g/L) ($\bar{x}\pm s$)	36.1 ± 6.9	39.7 ± 5.2	0.08	-
Total protein (g/L) ($\bar{x}\pm s$)	70.9 ± 8.6	70.8 ± 4.9	0.98	-
PCT >0.5 ng/ml [cases (%)]	0 (0.0)	13 (86.6)	<0.001	-
Eight types of respiratory pathogens [cases (%)]	-	-	-	-
*Legionella pneumophila*	0 (0.0)	0 (0.0)	-	-
*Mycoplasma pneumoniae*	18 (37.5)	5 (33.3)	0.77	1.20 (0.35,4.07)
*Chlamydia pneumoniae*	5 (10.4)	1 (6.6)	0.67	1.63 (0.19,14.12)
*Rickettsia burnetii*	0 (0.0)	0 (0.0)	-	-
*Influenza*	-	-	-	-
*Influenza A virus* IgM	3 (6.2)	2 (13.3)	0.38	0.43 (0.07,2.63)
*Influenza B virus* IgM	4 (8.3)	1 (6.6)	0.83	1.27 (0.14,11.51)
*Parainfluenza virus*	15 (31.2)	7 (46.6)	0.27	0.52 (0.16,1.70)
*Respiratory syncytial virus*	0 (0.0)	0 (0.0)	-	-
*Adenovirus*	0 (0.0)	0 (0.0)	-	-
M. tuberculosis etiological examination [cases (%)]	-	-	-	-
Acid—fast bacilli smear	6 (12.5)	1 (contaminated)	-	-
M. tuberculosis culture	3 (6.2)	0 (0.0)	-	-
M. tuberculosis molecular microbiology	20 (41.6)	1 (contaminated)	-	-
Fungal culture [cases (%)]	0 (0.0)	0 (0.0)	-	-
Positive pathogenic bacteria [cases (%)]	0 (0.0)	7 (46.6)	-	-
Positive blood IGRA	46 (95.8)	0 (0.0)	-	-
PPD >10 mm	28 (58.3)	0 (0.0)	-	-

hs-CRP, High-sensitivity C-reactive protein; PCT, Procalcitonin; ALT, Alanine aminotransferase; AST, Aspartate aminotransferase; Blood IGRA, Interferon-gamma release assay; PPD, Purified protein derivative of tuberculin; -, Not statistically analyzed.

In the control group, 7 children had positive pathogenic bacteria detection (3 cases with positive pathogenic bacteria culture and 5 cases with positive high-throughput sequencing), and the pathogenic bacteria involved were 2 cases of *Streptococcus pneumoniae*, 2 cases of *Pseudomonas aeruginosa*, 1 case of *Acinetobacter baumannii*, 1 case of *Klebsiella pneumoniae*, 1 case of *Serratia marcescens*, and 1 case of *Moraxella catarrhalis,* In the control group, one case exhibited a positive sputum smear for acid-fast bacilli, and another case had a positive M. tuberculosis molecular microbiology result. Based on clinical context, these specimens were comprehensively judged to be contaminated.

### 3.4 Comparison of imaging examination results between the two groups

No statistically significant differences were observed for cavity and pleural effusion on chest CT; however, all other features differed significantly between the groups (*P* < 0.05). The specific results of the chest CT comparison are shown in Table 4.

**Table 4:** Comparison of chest CT imaging features between the PTB group and the control group [cases (%)].

Imaging examinations	PTB group (*n* = 48)	Control group (*n* = 15)	*P*	OR(95%CI)
Cavity	9 (18.7)	1 (6.6)	0.48	3.16 (0.38, 26.10)
Pulmonary consolidation	17 (35.4)	9 (60.0)	0.05	0.36 (0.12, 1.08)
Fine linear/reticular opacities	30 (62.5)	2 (13.3)	<0.001	10.50 (2.20,50.06)
Patchy shadows with blurred edges	20 (41.6)	11 (73.3)	0.03	0.28 (0.08, 0.94)
Multiple pulmonary nodules	47 (97.9)	3 (20.0)	<0.001	235.00 (28.60, 1930.00)
Lymphadenopathy (hilar/mediastinal/axillary)	46 (95.8)	4 (26.6)	<0.001	69.00 (17.10,280.00)
Pleural effusion	6 (12.5)	1 (6.6)	0.88	1.98 (0.23, 16.87)
Calcification	35 (72.9)	1 (6.6)	<0.001	35.00 (4.40, 278.00)

Within the PTB group, 20 patients were identified as having concurrent TBTB, and 7 patients were diagnosed with concurrent CNS-TB. The specific subtypes and their respective frequencies among these complications are detailed in Table 5.

**Table 5:** Subgroup analysis of children with PTB complicated by TBTB or CNS-TB: findings from bronchoscopy and cranial MRI [cases (*n* %)].

Items	Cases (*n* %)
Bronchoscopic findings of TBTB	-
Inflammatory infiltration type	1 (2.0)
Ulcerative necrosis type	4 (8.3)
Granulation proliferation type	4 (8.3)
Lymph node fistula type	10 (20.8)
Scar stricture type	1 (2.0)
Cranial MRI findings of CNS-TB	-
Meningeal type	5 (10.4)
Cerebral parenchymal type	1 (2.0)
Mixed type	2 (4.1)

-, Not statistically analyzed; TBTB, Tracheobronchial tuberculosis; CNS-TB, Central nervous system tuberculosis.

### 3.5 Robust predictive model

Univariate analysis revealed that 14 independent variables were statistically significant. Following screening based on clinical significance, seven independent variables were included: Fine linear/reticular opacities, Patchy shadows with blurred edges, Multiple nodules, Lymphadenopathy(hilar/mediastinal/axillary), Calcification, History of tuberculosis exposure, Fever with chills.

Based on Lasso regression, two variables were included in the final model: multiple pulmonary nodules, lymphadenopathy(hilar/mediastinal/axillary).The results indicated that all two variables were independent influencing factors for distinguishing pediatric PTB from pneumonia initially suspected of PTB (all *P* < 0.05).The specific data are shown in Table 6.

**Table 6:** Multivariable firth penalized logistic regression model for the differentiation between pediatric PTB and pneumonia initially suspected of PTB.

Variables	Coefficient (*β*)	Standard error (SE)	OR (Bootstrap 95%CI)	*P*
Intercept	−4.61	0.82	0.00 (0.00,0.05)	*P* < 0.001
Multiple pulmonary nodules	5.62	0.83	276.18 (55.39,1430)	*P* < 0.001
Lymphadenopathy (hilar/mediastinal/axillary)	4.82	1.13	124.21 (10.58,867.35)	*P* < 0.001

The combined model achieved an AUC of 0.99 (95% CI: 0.97,1.00), with 100% specificity and 93.75% sensitivity, The data are shown in Table 7.

**Table 7:** Diagnostic performance of the combined predictive model and individual imaging indicators for differentiating pediatric PTB from pneumonia initially suspected of PTB.

Diagnostic model	AUC (95% CI)	Optimal cut-off	Sensitivity (%)	Specificity (%)	Accuracy (%)
Combined Model	0.99 (0.97,1.00)	0.40	93.75	100.00	95.24
Multiple pulmonary nodules	0.89 (0.81,0.97)	0.78	97.92	80.00	90.48
Lymphadenopathy (hilar/mediastinal/axillary)	0.85 (0.75,0.95)	0.69	95.83	73.33	87.30

AUC, Area under the curve; The combined model includes both “Multiple pulmonary nodules” and “Lymphadenopathy(hilar/mediastinal/axillary)” as predictors; Optimal cut-off was determined by maximizing Youden's index (J = Sensitivity + Specificity−1).

## 4 Discussion

PTB continues to pose a severe global public health threat, with a persistent and challenging epidemiological situation. According to the *Global Tuberculosis Report 2025* released by the WHO, an estimated 10.7 million individuals developed tuberculosis worldwide in 2024. Children accounted for 12% of these cases, equivalent to approximately 1.2 million new pediatric tuberculosis patients. Although tuberculosis is preventable and treatable, it led to around 1.23 million global deaths in 2024, including nearly 200 000 children (6). Due to immature immune function, children are more vulnerable to systemic dissemination after infection with M. tuberculosis. In regions with limited medical resources, the diagnosis of pediatric PTB faces prominent difficulties. Delayed diagnosis and treatment often result in disease progression and poor clinical outcomes (7). Even though tuberculosis is curable, missed or delayed diagnosis still exposes a large number of children to life-threatening conditions. This study compared the clinical characteristics between children with diagnosed PTB and those initially suspected of PTB but ultimately diagnosed with pneumonia, aiming to provide reliable evidence for the early differential diagnosis of pediatric PTB in clinical practice.

### 4.1 Key findings of pediatric PTB

The significant association between PTB and a history of tuberculosis contact confirms the fundamental epidemiological features of the disease. Previous research by Xu LL et al. demonstrated that a history of tuberculosis exposure (*OR* = 3.177, 95% CI: 1.345,7.452) serves as a risk factor for severe pediatric tuberculosis (8). These findings reinforce the indispensable role of detailed contact history collection in the diagnostic evaluation of children with suspected PTB. In the study, children in the PTB group had a longer pre-admission symptom duration and higher rates of prior antibiotic and corticosteroid use. This phenomenon may be attributed to initial misdiagnosis of PTB as pneumonia at primary medical institutions. Owing to the non-specific and overlapping clinical manifestations of PTB and common pneumonia (9), non-specialized clinicians often prescribe empirical antibacterial or corticosteroid treatment. Antibiotics are ineffective against M. tuberculosis. Corticosteroids can mask inflammatory symptoms and aggravate infection via immunosuppression, further delaying definitive diagnosis. In addition, the insidious onset and mild systemic symptoms observed in some children with PTB fail to arouse adequate attention from caregivers, which also leads to delayed medical seeking.

PCT differed significantly between the two groups in this study. However, PCT cannot be used as an independent diagnostic indicator, and clinical decision-making should not rely solely on this marker, as its level is affected by multiple confounding factors, including pre-admission antibiotic use, blood sampling time, disease progression, and control group selection. Accordingly, the between-group difference in PCT levels alone is insufficient for the differential diagnosis of pediatric PTB and pneumonia. In addition, the proportion of children with elevated ALT or AST was lower in the PTB group than in the pneumonia control group. This discrepancy may be attributed to case selection; therefore, it cannot be used as a reliable indicator for differential diagnosis.

It has been reported that pulmonary tuberculosis in children is prone to extrapulmonary complications (10). In the present cohort, some children with PTB also presented with concurrent TBTB and CNS-TB. This susceptibility is attributed to the immaturity of the pediatric immune system, which allows M. tuberculosis more readily breach the pulmonary barrier and disseminate systemically. Therefore, in clinical practice, vigilance for extrapulmonary manifestations should be maintained in children diagnosed with or highly suspected of PTB, in order to avoid missed diagnoses of extrapulmonary disease.

### 4.2 Diagnostic challenges and the value of imaging features

It is worth noting that in this study, the positive rates of acid-fast bacilli smear, rapid M. tuberculosis culture, and M. tuberculosis molecular testing in the PTB group were all low. This finding is consistent with previous studies (11) and lower than that reported in adult PTB (12). This phenomenon is closely associated with the inherent challenges in etiological detection of pediatric PTB. Children, especially infants, have not yet developed a mature cough reflex, making sputum collection difficult. In addition, the bacterial load in children infected with Mycobacterium tuberculosis is usually lower than that in adults. These factors collectively result in a low positive rate of etiological detection for pediatric PTB, which increases the difficulty of clinical diagnosis. Therefore, etiological detection results alone cannot be relied upon for the diagnosis of pediatric PTB. A comprehensive assessment is required, integrating medical history, clinical manifestations, imaging examinations, and other laboratory indicators, to improve the diagnostic accuracy.

The finding that multiple pulmonary nodules serve as a strong predictive factor in this study is anatomically and immunologically plausible. Pediatric PTB typically arises from primary infection, characterized by hematogenous dissemination of M. tuberculosis. This process induces the formation of multiple pulmonary granulomatous lesions (tubercles), which present as miliary or non-miliary nodules on chest CT (13, 14). Such imaging features differ markedly from those of pneumonia. Pneumonia more commonly manifests as lobar or segmental consolidation and patchy alveolar infiltrates, which explains the higher prevalence of patchy shadows with blurred margins observed in the control group.

These results are consistent with previous evidence. Tomà P et al. also described a high prevalence of nodular lesions in children with tuberculosis (15). Similarly, Wang B et al. summarized that typical CT manifestations of pediatric PTB include centrilobular nodules and mediastinal lymphadenopathy (16). The high prevalence of this specific imaging feature in the PTB cohort further confirms its important diagnostic significance.

Similarly, the high prevalence of hilar/mediastinal/axillary lymphadenopathy and its elevated diagnostic odds ratio represent a hallmark of pediatric tuberculosis. The immature immune system in children often mounts a pronounced lymphoid response to M. tuberculosis infection, resulting in prominent lymph node enlargement. A study by Mehrian P et al. on radiographic features of childhood tuberculosis also identified lymphadenopathy as one of the most common findings (17). The present data quantitatively confirm its discriminative power, which is consistent with the clinical teaching that significant adenopathy in a child with pulmonary infiltrates should raise a high index of suspicion for tuberculosis.

### 4.3 Model interpretation and integrated diagnostic approach

The model integrating the features of “multiple pulmonary nodules” and “lymphadenopathy” exhibited near-perfect discriminatory performance within the study cohort. This result suggests that in resource-constrained settings with limited access to molecular or advanced immunological testing, targeted chest CT assessment focusing on these two features may serve as a rapid and effective triage tool. Such an approach could facilitate earlier presumptive diagnosis of pediatric PTB, encourage more intensive efforts to obtain microbiological confirmation, and support the initiation of empirical treatment in severe cases while awaiting definitive diagnostic results, thereby potentially improving clinical outcomes.

However, the model's exceptional performance, particularly its 100% specificity, should be interpreted with caution. This likely reflects a “best-case scenario” derived from a cohort in which these two features were preselected as the most powerful discriminators. In real-world practice, atypical presentations do occur. Therefore, this model should not replace a comprehensive diagnostic approach but rather serve as a powerful adjunct within an integrated diagnostic algorithm that includes clinical assessment, exposure history, microbiological evidence, and available laboratory tests such as interferon-gamma release assays.

### 4.4 Limitations and future directions

The interpretation of these findings should be considered in light of several limitations. First, the single-center, retrospective study conducted at a specialized tuberculosis hospital may introduce referral bias, which likely enriched the PTB group with more typical cases. This may limit the generalizability of our results to primary or general healthcare settings. Second, the small sample size, particularly within the control group consisting of only 15 subjects, affects the precision of our estimates, as reflected in the wide confidence intervals, and increases the risk of overfitting the predictive model. Third, the exclusion of children with comorbidities or immunodeficiencies created a relatively homogenous cohort, which may not be fully representative of the broader patient population, especially given that such children are at higher risk for both severe pneumonia and disseminated tuberculosis.

## 5 Conclusion

In conclusion, this study identified a clinical-radiological signature that may be helpful in distinguishing pediatric PTB and pneumonia initially suspected of PTB. Beyond reaffirming the importance of exposure history, this study provided evidence for the discriminatory value of two specific chest CT features: multiple pulmonary nodules and hilar/mediastinal/axillary lymphadenopathy. A model based on these features demonstrated notable diagnostic potential. Although external validation of these findings is still needed, they underscore the critical role of thorough chest CT interpretation in resolving this common diagnostic dilemma. Clinicians should integrate these potent imaging clues with epidemiological and clinical data to accelerate accurate diagnosis and timely initiation of appropriate therapy for children with PTB.

## Data Availability

The original contributions presented in the study are included in the article/Supplementary Material, further inquiries can be directed to the corresponding author.
